# Sex-specific gene expression and weighted co-expression network analysis suggest distinct sex-specific molecular signatures in acutely suicidal MDD-patients without somatic comorbidities

**DOI:** 10.3389/fgene.2025.1653768

**Published:** 2025-10-03

**Authors:** Michael Fritz, Karlheinz Holzmann, Silvia Castany, Heidrun Haas, Christian Montag, David Engblom, Judith Streb, Manuela Dudeck

**Affiliations:** ^1^ Department of Forensic Psychiatry and Psychotherapy, Ulm University, Ulm, Germany; ^2^ Core Facility Genomics, Ulm University, Ulm, Germany; ^3^ Center for Social and Affective Neuroscience, Department of Biomedical and Clinical Sciences, Linköping University, Linköping, Sweden; ^4^ Department of Molecular Psychology, Ulm University, Ulm, Germany; ^5^ Centre for Cognitive and Brain Sciences, Institute of Collaborative Innovation, University of Macau, Macau SAR, China; ^6^ Department of Computer and Information Science, Faculty of Science and Technology, University of Macau, Macau SAR, China; ^7^ Department of Psychology, Faculty of Social Sciences, University of Macau, Macau SAR, China

**Keywords:** depression, suicidality, sex-specificity, gene expression, microarray, network analysis, WGCNA

## Abstract

**Introduction:**

Major depressive disorder (MDD) is a debilitating psychiatric disorder and is strongly associated with suicidal ideation and acute suicidality. While sex differences are evident across nearly all stages of depression, sex-specific mechanisms in acute suicidality remain not fully understood. This gap is notable given that women are twice as likely as men to develop depression, show earlier onset and greater symptom severity, and account for two-thirds of suicide attempts, whereas men have higher suicide completion rates. At the molecular level, sex differences also influence pharmacological treatment response, yet the biological mechanisms underlying these disparities remain not fully understood.

**Methods:**

In an exploratory approach, we investigated genome-wide gene expression changes in peripheral blood from 14 acutely suicidal patients with MDD (seven females, seven males) without comorbid somatic conditions, compared with sex-matched healthy controls. Gene expression profiles, generated using Affymetrix microarrays, were corrected for multiple testing and further examined through Gene Ontology enrichment, Gene Set Enrichment, Weighted Gene Co-expression Network, and Protein–Protein Interaction analyses.

**Results/Discussion:**

When analyzed as a combined group, suicidal MDD patients exhibited 87 differentially expressed genes (DEGs). However, stratification by sex revealed 665 DEGs in females, whereas no significant DEGs were detected in males. These findings, validated through pathway- and network-level analyses, suggest that previous studies pooling male and female MDD patients may have overlooked sex-specific effects. Nevertheless, given the small group number of patients, it cannot be excluded that the absence of DEGs in males may be due to a coincidental genetic profile of the group. Larger confirmatory studies, or re-analyses of existing datasets with sex-specific stratification, are therefore essential. In female suicidal MDD patients, both single-gene and pathway-oriented analyses highlighted immune and inflammatory processes, particularly the NF-κB pathway, consistent with prior evidence and pointing to additional targets such as tumor necrosis factor–alpha inducible protein 6.

**Conclusion:**

Collectively, these findings underscore the critical importance of sex-specific molecular research in acutely suicidal MDD patients and may inform the development of more targeted therapeutic approaches.

## Introduction

Major Depressive Disorder (MDD) is a debilitating mental health condition affecting millions worldwide. As of 2020, the World Health Organization (WHO) ranked MDD as the second leading cause of disease burden and disability, projecting it to take the top position by 2030 (Zhang et al., 2024b). Despite extensive research, MDD remains challenging to diagnose, treat, and prevent. Around 70% of patients fail to achieve full remission after an initial 12-week pharmacological treatment (LeGates et al., 2019), and up to 30% are classified as treatment-resistant after two failed pharmacological treatments (Moderie et al., 2022). This resistance may stem from the complex interplay of biological, environmental, and genetic factors influencing the disorder’s onset and progression.

In its most severe form, MDD can lead to suicide or suicide attempts, with MDD, schizophrenia, bipolar disorder, and substance use disorder being the four psychiatric illnesses, which are the leading underlying causes of suicide-related deaths (Shoib and Kim, 2019; Wilk et al., 2023).

An critical yet underexplored factor in course and progression of MDD and its most severe outcome–suicidality–is biological sex. Women are diagnosed with depression about twice as often as men (Brody et al., 2018), and across cultures they attempt suicide two to three times more frequently (Weissman et al., 1999). One explanation for the difference in suicide attempts may be the reported observation that women tend to show greater symptom severity, earlier onset, and longer depressive episodes (Marcus et al., 2005). This may, in part, reflect sex-dependent differences in coping styles related to MDD symptomatology. Women have been shown to employ less cognitive reframing and to report higher levels of self-blame (Kelly et al., 2008). Such cognitive patterns may contribute to increased perceptions of helplessness and hopelessness. Paradoxically, despite worse MDD symptomatology and disease progression, higher levels of self-blame, as well as more suicide attempts amongst women, men have higher rates of completed suicide (Branney and White, 2008). Some studies attempt to explain this conundrum by proposing sex-specific behavioral manifestations of depression and suicidality, suggesting that men may express suicidal tendencies through behaviors such as risk-taking, which may not be sufficiently assesses clinically (Oliffe et al., 2016). However, this hypothesis remains inconclusive, as demonstrated by Soravia et al. (2024).

Sex differences also extend to treatment response. Men generally respond better to tricyclic antidepressants, whereas women are more likely to benefit from selective serotonin reuptake inhibitors (SSRIs) [for review, see (LeGates et al., 2019)]. Collectively, these differences suggest sex-specific biological mechanisms in patients with MDD as well as MDD-driven suicidality, which are known to persist even at the gene expression level (Lopes-Ramos et al., 2020), yet remain underexplored.

In this context, blood-based gene expression profiling has emerged as a promising tool for identifying molecular signatures associated with psychiatric conditions (Reay et al., 2022). Blood is not only easily accessible and minimally invasive to sample but also reflects systemic biological changes, including those relevant to brain function and mood regulation. For instance, the progression of neurodegeneration is reflected in both blood and brain gene expression (Iturria-Medina et al., 2020). However, scientific caution is necessary when interpreting blood gene expression results, as they do not perfectly mirror brain expression and can vary depending on the sample source (e.g., whole blood, tissue, or immune cells) as well as the specific brain region being studied (Sullivan et al., 2006).

Nonetheless, in the study of MDD, blood gene expression and protein analyses are gaining traction, identifying immunological genes and protein structures both preclinically and clinically as potential biomarkers for diagnosis and treatment response [for review, see (Mariani et al., 2021)]. However, research on acute suicidality remains scarce, with only three studies demonstrating a correlation between interleukin-6 protein concentrations in cerebrospinal fluid (Lindqvist et al., 2009) and blood plasma (Sun et al., 2023b; Castillo-Avila et al., 2022; Wang et al., 2025) and the severity of suicidal ideation. A major limitation of these studies, however, is the absence of group comparisons according to biological sex within the acutely suicidal group, which may obscure potential sex-specific biological mechanisms underlying suicidality.

Studies utilizing large-scale gene array approaches in the context of suicidality are, to the best of our knowledge, equally scarce and share similar methodological limitations, including a lack of sex differentiation (Sun et al., 2024; Daskalakis et al., 2024; Wang et al., 2025).

Hence, the present study aims to address these limitations by investigating sex-specific blood plasma-derived gene expression patterns in acutely suicidal patients with MDD, excluding those with additional physiological or psychiatric diagnoses known to affect gene expression. This approach allows for a more focused and unbiased analysis of depression related acute suicidality on a whole-genome level, but reduces the numbers of participants per group.

By identifying genes, pathways, and regulatory networks that differ between suicidal male and female patients with MDD, this research seeks to deepen our understanding of the biological basis of sex-specific manifestations of MDD driven suicidality. Ultimately, these insights can contribute to the development of more precise and individualized therapeutic strategies for this high-risk population.

## Materials and methods

### Patients

From 2021 to 2023, a total of 14 Caucasian patients (seven male and seven female) age 18 or older with acute suicidal ideation were recruited from the Clinic for Psychiatry and Psychotherapy II at Ulm University, located at the district hospital in Günzburg, Bavaria, Germany. The study was conducted in accordance with the Declaration of Helsinki and received approval from the ethical review board of the Bavarian State Medical Association (Nr. 21007). Patient recruitment and blood collection were performed within the first 24 h of hospital admission by two independent doctoral students. For the purposes of this study, “acute suicidal ideation” was defined as the presence of active suicidal thoughts accompanied by an acute intent to commit suicide, with symptom severity comparable to ICD-10 diagnosis F32.2 and requiring inpatient treatment. Patients presenting with such an acute symptom severity were identified and referred to the doctoral students by on-duty clinical staff.

Inclusion criteria comprised ICD-10 diagnoses F32, F33, F34, F38, or F39, as well as sufficient proficiency in German.

Exclusion criteria included any diagnosis within the schizophrenia spectrum, intellectual disability, bipolar disorder, eating disorders, substance use disorders (SUDs), as well as cancer, chronic inflammatory diseases, epilepsy, diabetes, coronary heart disease, obesity, and pregnancy. Although posttraumatic stress disorder (PTSD) was not explicitly listed as an exclusion criterion, none of the participants had a documented PTSD diagnosis in their medical records. With regard to personality disorders, only one male and one female participant had a diagnosis of borderline personality disorder. Given the very low numbers and the equal distribution across sexes, it is unlikely that borderline personality disorder would account for the observed differences, and it was therefore not included as a covariate in the analysis.

Additionally, 14 sex- and ethnicity-matched healthy controls were recruited via public notices, social media advertisements, and personal contacts, following the same exclusion criteria. For specific sociodemographic details, see Table 1. In Table 1, the term “*smoker*” refers to participants who answered “yes” when asked whether they consume cigarettes. The exact number of cigarettes smoked per day was not assessed.

**TABLE 1:** Sociodemographic suicidal and control group details.

Group details	Male	Female	Statistics
	*M (SD)/n*	*M (SD)/n*	
Acutely suicidal patients	7	7	
Age (years)^a^	41.4 (15,0)	43.1 (15,0)	*t* (12) = −0.213, *p* = 0.835, Cohen’s *d* = −0.114
Highest educational degree^a^			Fishers exact test, *p* = 0.848, Cramer *V* = 0.434
None	1	1	
Middle School Diploma	3	2	
Secondary School Diploma	1	2	
High School Diploma	2	0	
Diagnoses^a^			Fishers exact test, *p* = 1.000, Cramer *V* = 0.076
Depression	6	4	
with borderline personality disorder	1	1	
Durations of depressive symptoms (years)^a^	8.7 (14.9)	13.8 (18.3)	*z* = −0.763 *p* = 0.446, Cohen’s *d* = 0.473
Previous suicide attempts^a^	4	2	Fishers exact test, *p* = 1.000, Cramer *V* = 0.169
Body Height (cm)^a^	175.4 (6.3)	165.4 (4.5)	*t* (10) = 3.033, *p* = 0.013, Cohen’s *d* = 1.776
Body Weight (kg)^a^	86.4 (15.2)	73.0 (9.7)	*t* (10) = 1.731, *p* = 0.114, Cohen’s *d* = 1.013
Smoker^a^	3	2	Fishers exact test, *p* = 1.000, Cramer *V* = 0.069
Healthy controls	7	7	
Age (years)	31.9 (8.6)	30.4 (11.9)	*t* (12) = 0.258, *p* = 0.801, Cohen’s *d* = 0.138
Highest educational degree			Fishers exact test, *p* = 0.559, Cramer *V* = 0.316
None	—	—	
Middle School Diploma	—	—	
Secondary School Diploma	1	3	
High School Diploma	6	4	
Diagnoses (self-reported)	—	—	
Previous suicide attempts	—	—	
Body Height (cm)	179.9 (5,7)	166.3 (7.7)	*t* (12) = 3.749, *p* = 0.003, Cohen’s *d* = 2.004
Body Weight (kg)	80.1 (10.8)	61.9 (6.0)	*t* (12) = 3.908, *p* = 0.002, Cohen’s *d* = 2.089
Smoker	1	2	Fishers exact test, *p* = 0.559, Cramer *V* = 0.225

^a^ Missing values = 2, *M* = mean, *SD*, standard deviation, *n* = number of observations, *t* = *t*-value, *z* = *z*-value.

### RNA isolation and qRT-PCR

Blood samples were collected in PAXgene^®^ Blood RNA tubes and stored according to manufacturer’s protocol at −80 °C. RNA was then extracted using the MagNA Pure 96 Cellular RNA Large Volume Kit following the manufacturer’s instructions. RNA concentration and quality were assessed using a NanoDrop spectrophotometer (Thermo Fisher Scientific, Waltham, MA, United States), and only samples with an A260/A280 ratio greater than 1.8 were included in the experiment. Complementary DNA (cDNA) was synthesized using the High-Capacity cDNA Reverse Transcription Kit (Applied Biosystems, Foster City, CA, United States). Quantitative PCR was performed using the TaqMan Gene Expression Master Mix (Applied Biosystems) with the following TaqMan gene expression assays: Interleukin 1 beta (Il1b; Hs01555410_m1), Interferon gamma receptor 2 (Ifgnr2; Hs00194264_m1), and TNF alpha-induced protein 6 (Tnfai6; Hs00200180_m1). Glyceraldehyde 3-phosphate dehydrogenase (Gapdh; Hs02786624_g1) was used as an endogenous control. All three genes were selected based on their ranking among the most differentially expressed genes. Reactions were conducted using the Real-Time 7900 Fast system (Applied Biosystems). Relative quantification was performed using the 2^-ΔΔCT method, and gene expression changes were expressed as fold change relative to the corresponding control group (male or female healthy volunteers).

### Microarray analysis

Microarray analyses were performed using 200 ng total RNA as starting material and 5.5 µg ssDNA per hybridization (GeneChip Fluidics Station 450; Affymetrix, Santa Clara, CA). The total RNAs were amplified and labeled following the Whole Transcript (WT) Sense Target Labeling Assay (http://www.affymetrix.com). Labeled ssDNA was hybridized to Human Clariom S Affymetrix GeneChip arrays (Affymetrix, Santa Clara, CA). The chips were scanned with an Affymetrix GeneChip Scanner 3000 and subsequent images analyzed using Affymetrix^®^ Expression Console™ Software (Affymetrix). A transcriptome analyses was performed using BRB-ArrayTools developed by Dr. Richard Simon and BRB-ArrayTools Development Team (http://linus.nci.nih.gov/BRB-ArrayTools.html), as published previously (Zoller et al., 2017). Raw feature data were normalized and log_2_ intensity expression summary values for each probe set were calculated using robust multiarray average (Irizarry et al., 2003).

### Filtering

Genes showing minimal variation across the set of arrays were excluded from the analysis. Genes whose expression differed by at least 1.5-fold from the median in at least 20% of the arrays were retained.

### Class comparison

We identified genes that were differentially expressed among the two classes using a two-sample t-test. Genes were considered statistically significant if their p value was less than 0.05 and displayed a fold change between the two groups of at least 1.5-fold. We used the Benjamini and Hochberg correction to provide 90% confidence that the false discovery rate was less than 10% (Benjamini and Hochberg, 1995). The false discovery rate is the proportion of the list of genes claimed to be differentially expressed that are false positives.

### Class prediction

We developed models for utilizing gene expression profile to predict the class of future samples. We developed models based on the Compound Covariate Predictor (Radmacher et al., 2002), Diagonal Linear Discriminant Analysis (Dudoit et al., 2002), Nearest Neighbor Classification (Dudoit et al., 2002), and Support Vector Machines with linear kernel (Ramaswamy et al., 2001). The models incorporated genes that were differentially expressed at the 0.001 significance level as assessed by the random variance t-test (Wright and Simon, 2003). We estimated the prediction error of each model using leave-one-out cross-validation (LOOCV) as described by (Simon et al., 2003). For each LOOCV training set, the entire model building process was repeated, including the gene selection process. We also evaluated whether the cross-validated error rate estimate for a model was significantly less than one would expect from random prediction. The class labels were randomly permuted and the entire LOOCV process was repeated. The significance level is the proportion of the random permutations that gave a cross-validated error rate no greater than the cross-validated error rate obtained with the real data. 1000 random permutations were used.

### Gene ontology analysis of differentially expressed genes

To identify associated biological processes, as defined by Gene Ontology annotation, we used the GoMINER analysis tool (Zeeberg et al., 2003). This package allows the automatic analysis of multiple microarrays and then integrates the results, across all of them, to find the GO categories that were significantly over- or under-represented.

### RNA-seq data analysis

Differential gene expression data were generated by downloading count data from the dataset GSE247998 (Sun et al., 2024), downloaded from Gene Expression Omnibus, and analyzed using the R-package Desq2 (Love et al., 2014) version 1.44 under R version 4.4. Genes with read counts below 10 were excluded.

### Gene set enrichment analysis

To investigate the biological pathways and functional gene sets enriched in the blood transcriptomic profiles of female patients with MDD and suicidal ideation, we performed Gene Set Enrichment Analysis (GSEA) using GSEA Version 4.3.3 (https://www.gsea-msigdb.org/gsea/index.jsp) and genesets Hallmark (H), curated genesets (C2) and immunologic genesets (C7) (Subramanian et al., 2005; Mootha et al., 2003) on the preprocessed gene expression data.

GSEA was run using the following parameters: gene set size filter of 15–500 genes, 1,000 permutations, and the weighted enrichment statistic. Statistical significance was determined based on a false discovery rate (FDR) q-value <0.25, as recommend in the documentation (https://docs.gsea-msigdb.org/#GSEA/GSEA_User_Guide/#interpreting-gsea-results).

### Weigthed gene coexpression network analysis (WGCNA)

In recent years, a powerful method for systematic analysis called weighted gene co-expression network analysis (WGCNA) has been widely applied in bioinformatic analysis of various diseases (Zhang and Horvath, 2005). The robust co-expression network is able to cluster genes with similar expression patterns into modules to identify the underlying biological functions. To identify gene co-expression modules associated with suicidal ideation, we used this method on the transcriptomic data using the WGCNA R-package (R version 4.3) developed by P. Langfelder, and S. Horvath (Langfelder and Horvath, 2008).

#### Data preprocessing for WGCNA analysis

Gene expression data were preprocessed by removing low-expression genes (lowest 30%) and retaining the top 50% genes based on variance expression variability. 7507 genes/probesets remained after filtering.

#### Network construction

A weighted adjacency matrix was calculated using a soft threshold power of ß = 12, determined using the scale-free topology criterion. The matrix was transformed into a Topological Overlap Matrix (TOM) to measure network connectivity. As parameters for the analysis we used: TOMType = “signed”, minModuleSize = 30, mergeCutHeight = 0.25.

#### Module-phenotype associations

Modules were correlated with clinical traits (sex, suicide attempt) using eigengene values as module representatives. Modules with significant associations (p < 0.05) were prioritized for downstream analysis. Hub gene identification was based on a gene significance >0.2 and a module membership >8 (Liang et al., 2020).

#### Functional annotation

Genes within significant modules were subjected to enrichment analysis to determine overrepresented biological processes and pathways using GSEA as well as STRING (see below).

### STRING analysis

STRING database 12.0 (Szklarczyk et al., 2023), https://string-db.org/) was used to identify possible protein-protein interactions between differentially regulated genes as well as between WGCNA hub genes. Default settings (Full STRING network; network edges: evidence; all active interaction sources and medium confidence) were used for both analyses.

## Results

### Gene expression analysis

We first analyzed male and female acutely suicidal patients with MDD together and identified 87 differentially expressed genes (91 probesets) (p-value <0.05, FDR < 0.1, |FC| > 1.5x), a threshold applied across all subsequent comparisons. The complete gene list is provided in Supplementary Table S1.

To determine whether transcriptional profiles could effectively distinguish suicidal patients with MDD from healthy controls, we applied several class prediction methods and observed a striking difference between male and female patients. In the male group, only two of seven algorithms (Compound Covariate Predictor and Diagonal Linear Discriminant Analysis) correctly predicted a maximum of 9 out of 14 samples. In contrast, three algorithms in the female group correctly predicted all samples, while the remaining four accurately classified 13 out of 14 samples. This result was further supported by a PCA analysis of the gene expression data.

**FIGURE 1:**
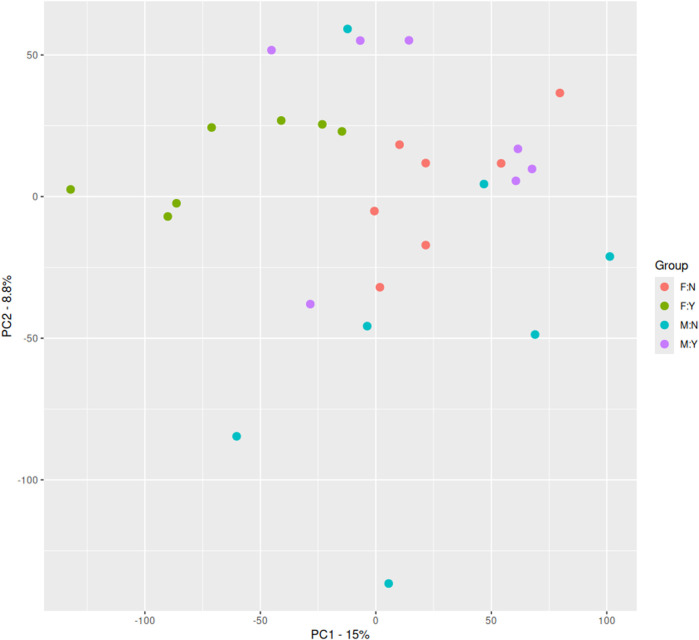
Principal component analysis of our microarray data. N = Female, healthy Control, F:Y = Female, Suicide Attempt, M:N = Male, healthy Control, M:Y = Male, Suicide Attempt.

As shown in Figure 1, there is only a clear separation between the female suicidal ideation group and their corresponding healthy control group, but not between the corresponding male subgroups. Based on these findings, we proceeded with sex-specific differential gene expression analyses.

This approach revealed no differentially expressed genes in suicidal men with MDD compared to healthy male controls. However, in women, we identified 671 differentially expressed probesets corresponding to 665 genes, with 304 downregulated and 367 upregulated. A list of the up- and downregulated genes is available in Supplementary Table S2.

To ensure these findings were not driven by differences between the male and female healthy control groups, we compared their gene expression profiles. This analysis yielded only 23 differentially expressed genes, all located on the sex chromosomes. Additionally, when comparing suicidal male and female MDD patients, we identified only 40 differentially expressed genes, 26 of which were XY-chromosomal (provided as Supplementary Table S3). These results strongly indicate that the observed gene expression differences are specifically associated with MDD-driven suicidality and sex-dependent to their respective healthy controls.

Gene Ontology enrichment analysis of the differentially expressed genes in suicidal women with MDD revealed significant enrichment in immune system and inflammatory pathways. The top 20 GO terms are presented in Supplementary Table S4.

### STRING analysis

**FIGURE 2:**
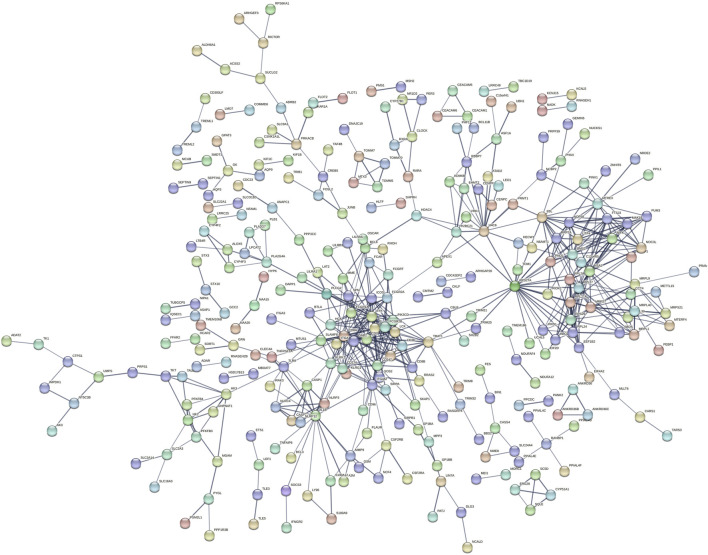
STRING analysis on the set of differentially regulated genes between female patients with suicide attempts and their respective healthy control group.

To identify and visualize potential protein-protein interactions within the set of differentially regulated genes in the female MDD patient group with acute suicidal ideations, we used the STRING database. The network as identified by STRING [Figure 2; Supplementary Figure S1 (high resolution)] revealed a complex network of interactions mainly centered around two clusters of genes consisting of immunological genes such as *SYK*, *FCGR1A* or *LCK* or genes involved in ribosomal biosynthetic processes such as *RPS24*, *RPL27a*, *NSA2* or *BMS1*.

### GSEA analysis

Gene Set Enrichment Analysis (GSEA) of the female gene expression data identified 12 significantly enriched gene sets (FDR < 0.25) when compared to the Hallmark gene sets (Table 2). The top three enriched pathways were TNFA signaling via NFKB, IL6-JAK-STAT3 signaling, and inflammatory response. Among the identified pathways, TNF-alpha Induced Protein 6 (*TNFAIP6*) emerged as the top-ranked gene in both TNFA signaling via NFKB and inflammatory response pathways. Other notably differentially expressed genes were Bestrophin 1 (*BEST1*), Protein Tyrosine Phosphatase Receptor Type r (*PTPRE*), Plasminogen Activator/Urokinase Receptor (*PLAUR*), Aquaporin 9 (*AQP9*), the inward-rectifying potassium channel KCJN2 and alpha-2-macroglobulin (*A2M*).

**TABLE 2:** Significant genesets within the Hallmark collection of MSigDB using the gene expression data from the female sample group.

Geneset	Size	ES	NES	NOM p-val	FDR q-val	FWER p-val
HALLMARK_TNFA_SIGNALING_VIA_NFKB	197	0.54	1.87	0.002	0.016	0.015
HALLMARK_IL6_JAK_STAT3_SIGNALING	87	0.54	1.76	0	0.039	0.062
HALLMARK_INFLAMMATORY_RESPONSE	199	0.47	1.68	0.004	0.058	0.125
HALLMARK_COMPLEMENT	199	0.44	1.66	0.008	0.055	0.148
HALLMARK_HYPOXIA	193	0.38	1.51	0.002	0.171	0.39
HALLMARK_ANGIOGENESIS	36	0.49	1.46	0.04	0.209	0.507
HALLMARK_APOPTOSIS	159	0.35	1.45	0.033	0.198	0.534
HALLMARK_EPITHELIAL_MESENCHYMAL_TRANSITION	197	0.38	1.44	0.044	0.176	0.535
HALLMARK_COAGULATION	138	0.38	1.38	0.063	0.249	0.699
HALLMARK_KRAS_SIGNALING_UP	198	0.33	1.37	0.038	0.228	0.705
HALLMARK_UV_RESPONSE_UP	155	0.28	1.36	0.009	0.228	0.735
HALLMARK_KRAS_SIGNALING_DN	195	0.35	1.35	0.05	0.225	0.756

**FIGURE 3:**
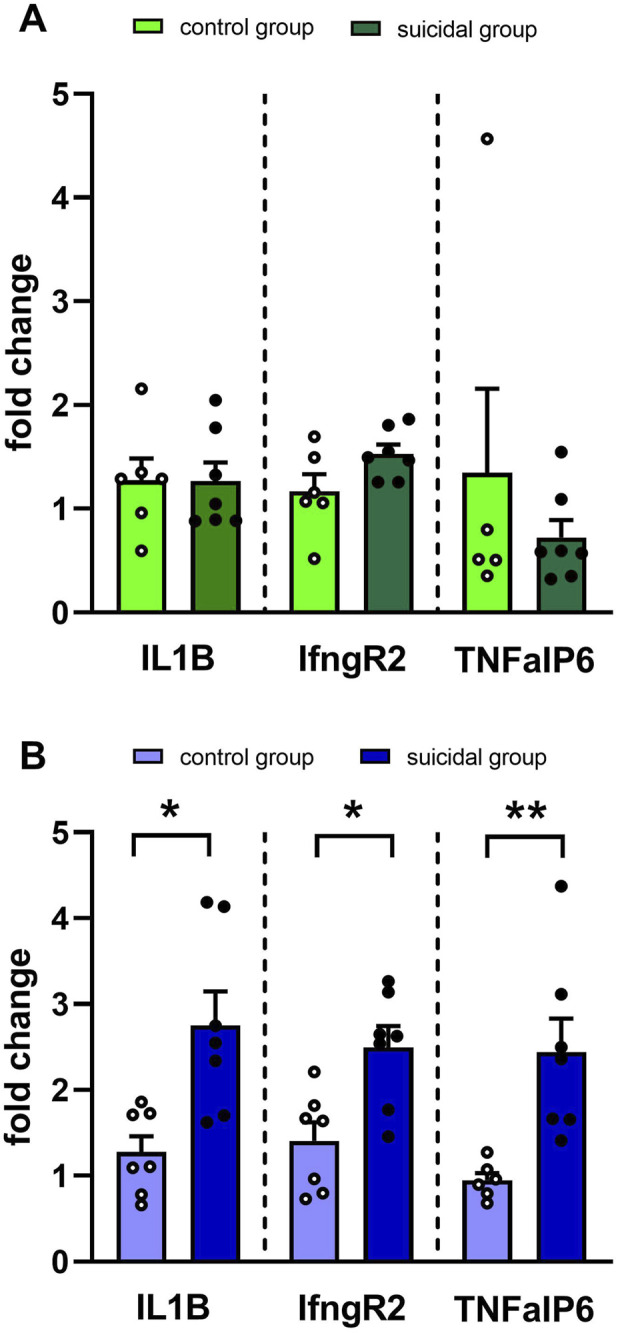
Validation of gene expression changes from selected differentially expressed genes in blood of male **(A)** and female **(B)** suicidal patients using qRT-PCR. Data are expressed as mean ± SEM. Gene expression changes are expressed as fold change versus the control group (Healthy individuals). Significance was determined with Student’s t-test, with *p < 0.05, **p < 0.01. IL1B = Interleukin 1-beta, IfngR2 = Interferon Gamma Receptor 2, TNFAiP6 = TNF-alpha Induced Protein 6.

Since TNFAIP6 was found to be significantly overexpressed in the suicidal group, it was together with Interleukin 1-beta (*IL1B*) and Interferon Gamma Receptor 2 (*IFNgR2*) (Fritz et al., 2018) selected for validation using quantitative PCR. Consistent with the initial array findings, all three were not upregulated in the male population. However, *IL1B*, *IFNgR2*, and *TNFAIP6* exhibited significant differential expression between suicidal women with MDD and their respective healthy controls (see Figure 3).

**FIGURE 4:**
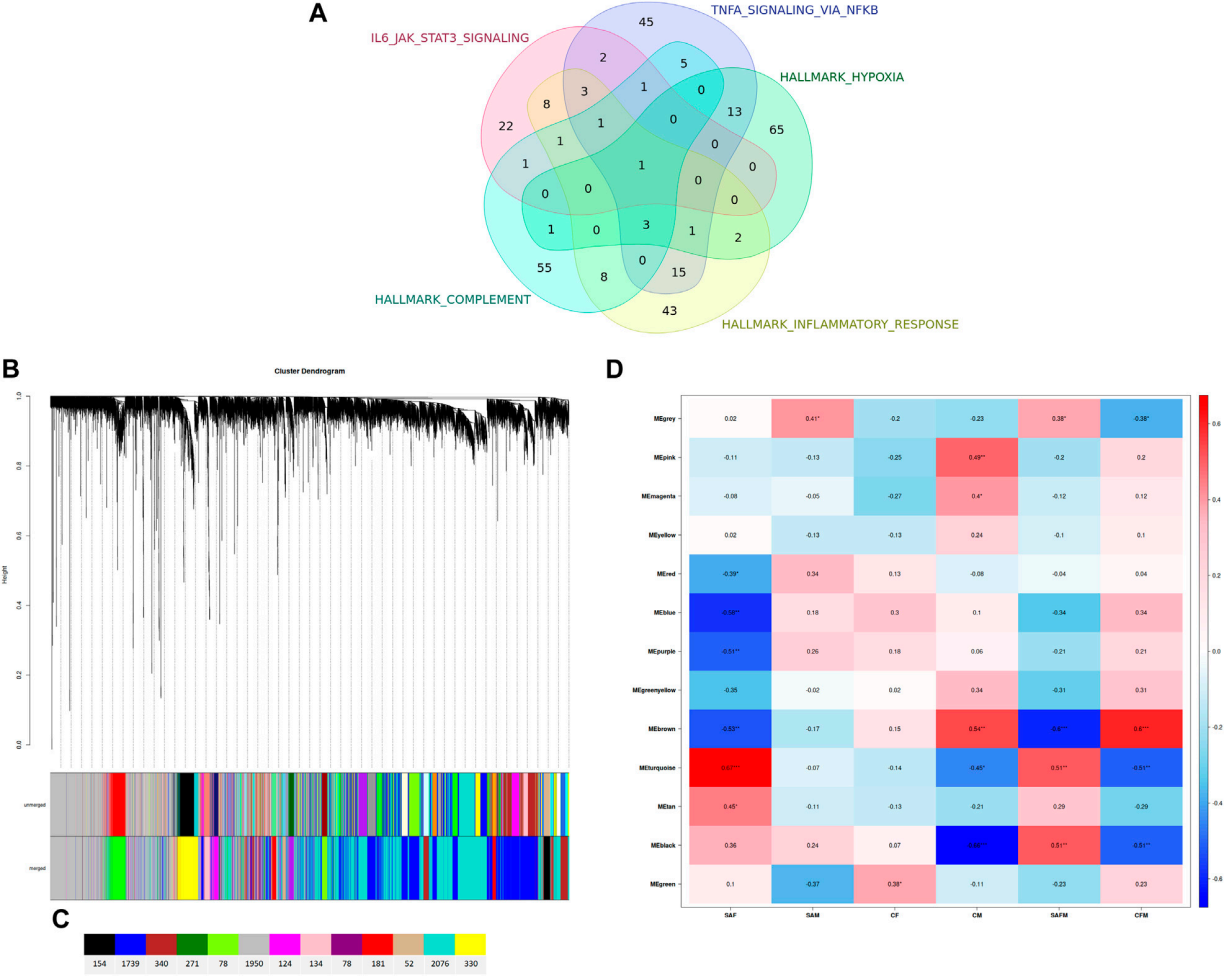
**(A)** Venn diagram illustrating the overlap of the leading-edge subset of genes (female dataset only) that are found within the top five enriched genesets of the Hallmark geneset. **(B)** Clustering dendrogram of all genes, with dissimilarities based on topological overlap. Each vertical line represents a single gene. Color designations assigned to the various modules within the coexpression network are shown below. **(C)** Number of genes assigned to the different coexpression networks. **(D)** Heatmap of the correlation between modules of genes and sample groups. This representation is a visual depiction of the Weighted Gene Co-expression Network Analysis (WGCNA). The heatmap reveals the correlation coefficient as well as the statistical significance of the data (*P ≤ 0.05, **P ≤ 0.01, ***P ≤ 0.001). Additionally, it indicates the direction of the correlation, whether positive (red) or negative (blue). The y-axis corresponds to the modules of genes grouped together according to their expression patterns. The x-axis denotes the sample group: SAF = Suicide Attempt, Female; SAM = Suicide Attempt, Male; CF = Healthy Control Female; CM = Healthy Control Male; SAFM = Suicide Attempt, Male and Female; CFM = Healthy Control Female and Male.

Given the overlap between genes in the top five GSEA-enriched pathways, we constructed a Venn diagram to illustrate shared gene expression patterns (Figure 4A; Table 3). Interleukin-6 (IL6) emerged as the top shared gene, present in all five pathways.

**TABLE 3:** Overlap of the leading-edge genes within the top 5 genesets of the Hallmark collection.

Gene	Number of occurences in the TOP 5 Hallmark genesets	TNFA signaling VIA NFKB	Hallmark inflammatory response	Hallmark hypoxia	Hallmark complement	IL6 JAK STAT3 signaling
IL6	5	X	X	X	X	X
F3	4	X	X	X	X	
IRF1	4	X	X		X	X
PLAUR	4	X	X	X	X	
SERPINE1	4	X	X	X	X	
CXCL1	3	X			X	X
IFNGR2	3	X	X			X
IL1B	3	X	X			X
KLF6	3	X	X	X		
PIK3R5	3		X		X	X
TLR2	3	X		X		X
ABCA1	2	X		X		
ADM	2		X	X		
BHLHE40	2	X		X		
BTG2	2	X	X			
CCL7	2		X			X
CCRL2	2	X	X			
CD14	2		X			X
CD55	2		X		X	
CEBPB	2	X			X	
CHST2	2		X	X		
CSF2	2	X				X
CSF3R	2		X			X
CXCL6	2	X	X			
DUSP1	2	X		X		
EHD1	2	X			X	
FOS	2	X		X		
FOSL2	2	X		X		
GNAI3	2		X		X	
GP1BA	2		X		X	
GRB2	2				X	X
ICAM1	2	X	X			
ICOSLG	2	X	X			
IER3	2	X		X		
IL18	2	X	X			
IL18R1	2		X			X
IL1A	2	X	X			
IL1R1	2		X			X
IL4R	2		X			X
IRF7	2		X		X	
IRS2	2	X		X		
ITGB3	2		X			X
KYNU	2	X			X	
LYN	2		X		X	
MXD1	2	X	X			
NAMPT	2	X	X			
NFIL3	2	X		X		
NFKBIA	2	X	X			
PDE4B	2	X	X			
PDGFB	2			X	X	
PFKFB3	2	X		X		
PLEK	2	X			X	
PPP1R15A	2	X		X		
PSEN1	2		X		X	
PTPRE	2	X	X			
RHOG	2		X		X	
SDC4	2	X		X		
SERPINB2	2	X			X	
SLC2A3	2	X		X		
SOCS3	2	X				X
TIMP1	2		X		X	
TNFAIP6	2	X	X			
TNFRSF1B	2		X			X
TNFRSF9	2		X			X
VEGFA	2	X		X		
ZFP36	2	X		X		

To further validate our sex-specific findings, we repeated the same analysis with the male dataset, especially since GSEA analysis is not limited to a set of differentially expressed genes that could be biased by filtering conditions on fold-change and p-value. In support of our sex-specific findings, not a single pathway was identified (FDR < of 0.25) in both the hallmark and curated dataset collection (C2) in contrast to 12 and 61 genesets in the female dataset.

### WGCNA analysis

To further explore the regulatory networks within our dataset, we performed weighted gene co-expression network analysis (WGCNA). Similar to GSEA, WGCNA is not restricted to a predefined set of differentially expressed genes but instead utilizes the entire gene expression dataset. After testing multiple candidate powers, we selected a soft threshold power of 12 based on the approximate scale-free topology criterion. This resulted in the identification of 13 distinct WGCNA modules (Figure 4B), with module sizes ranging from 52 to 2,076 genes (Figure 4C).

To identify modules significantly associated with suicidal ideations (correlation >0.5, p < 0.001), we correlated eigengenes with both sex and suicide attempt status. The resulting correlation plot (Figure 4D) again revealed a striking difference between the male and female MDD-patients with suicidal ideation. In contrast to the male group, where no highly significant correlations were observed, the female group exhibited a highly significant module (turquoise) with a strong correlation (r = 0.67, p < 0.001). Interestingly, when male and female data were combined in the analysis, the correlation strength for this module decreased (from r = 0.67 to r = 0.51) alongside its statistical significance (from p < 0.001 to p < 0.05). This further supports the necessity of analyzing male and female data separately in studies of gene expression related to suicidality.

**FIGURE 5:**
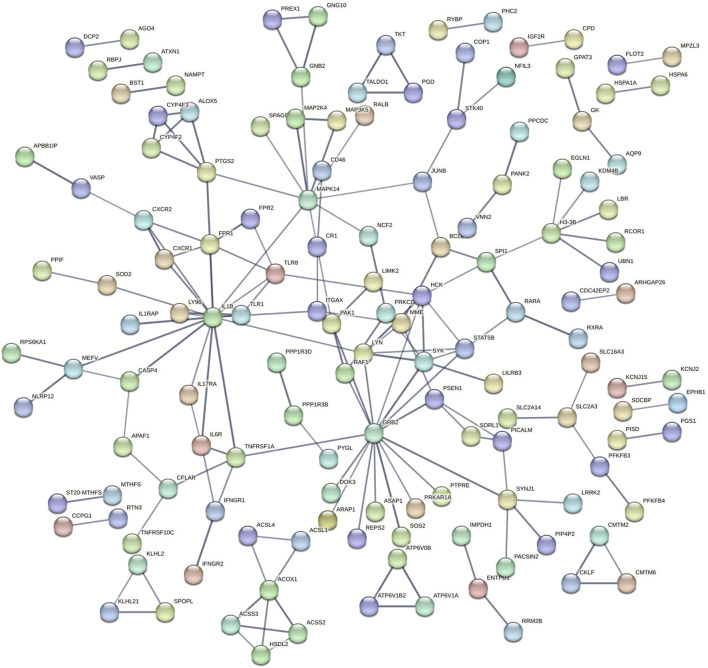
STRING analysis on the set of hub genes within the high significant co-expression network (“turquoise”) of the female sample group.

Next, we identified key regulatory genes, or “hub genes,” within the turquoise module using gene significance (GS > 0.3) and module membership (MM > 0.8) criteria (Liang et al., 2020). A total of 363 hub genes (Supplementary Table S8) were identified, which were then subjected to gene set enrichment analysis using the Hallmark collection. The results closely mirrored those from the GSEA of differentially expressed genes, with significant enrichment in TNF alpha signaling via NFKB, inflammatory response, and complement pathways (Table 4). To further investigate interactions between these hub genes, we conducted a STRING analysis. The resulting gene interaction network (Figure 5; Supplementary Figure S5) revealed two major central nodes: *IL1B* (inflammatory response) and *GRB2* (RAS-ERK signaling pathway).

**TABLE 4:** Significant genesets within the Hallmark collection of MSigDB using the hub genes from the WGCN analysis of the female sample group.

Gene set name	Genes in gene set (K)	Description	Genes in overlap (k)	k/K	p-value	FDR q-value
HALLMARK_TNFA_SIGNALING_VIA_NFKB	200	Genes regulated by NF-kB in response to TNF (GeneID = 7124)	18	0.09	1.47E−13	7.36E−12
HALLMARK_INFLAMMATORY_RESPONSE	200	Genes defining inflammatory response	16	0.08	2.03E−11	5.07E−10
HALLMARK_COMPLEMENT	200	Genes encoding components of the complement system, which is part of the innate immune system	15	0.075	2.15E−10	3.58E−09
HALLMARK_APOPTOSIS	161	Genes mediating programmed cell death (apoptosis) by activation of caspases	11	0.0683	1.46E−07	1.82E−06
HALLMARK_HEME_METABOLISM	200	Genes involved in metabolism of heme (a cofactor consisting of iron and porphyrin) and erythroblast differentiation	10	0.05	8.95E−06	6.81E−05
HALLMARK_MTORC1_SIGNALING	200	Genes upregulated through activation of mTORC1 complex	10	0.05	8.95E−06	6.81E−05
HALLMARK_IL6_JAK_STAT3_SIGNALING	87	Genes upregulated by IL6 (GeneID = 3569) via STAT3 (GeneID = 6774), e.g., during acute phase response	7	0.0805	9.53E−06	6.81E−05
HALLMARK_XENOBIOTIC_METABOLISM	200	Genes encoding proteins involved in processing of drugs and other xenobiotics	9	0.045	5.71E−05	3.57E−04
HALLMARK_PEROXISOME	104	Genes encoding components of peroxisome	6	0.0577	2.65E−04	1.36E−03
HALLMARK_PI3K_AKT_MTOR_SIGNALING	105	Genes upregulated by activation of the PI3K/AKT/mTOR pathway	6	0.0571	2.79E−04	1.36E−03
HALLMARK_IL2_STAT5_SIGNALING	199	Genes upregulated by STAT5 in response to IL2 stimulation	8	0.0402	3.16E−04	1.36E−03
HALLMARK_ADIPOGENESIS	200	Genes upregulated during adipocyte differentiation (adipogenesis)	8	0.04	3.27E−04	1.36E−03
HALLMARK_TGF_BETA_SIGNALING	54	Genes upregulated in response to TGFB1 (GeneID = 7040)	4	0.0741	1.14E−03	4.38E−03
HALLMARK_ALLOGRAFT_REJECTION	200	Genes upregulated during transplant rejection	7	0.035	1.66E−03	5.18E−03
HALLMARK_INTERFERON_GAMMA_RESPONSE	200	Genes upregulated in response to IFNG (GeneID = 3458)	7	0.035	1.66E−03	5.18E−03
HALLMARK_KRAS_SIGNALING_UP	200	Genes upregulated by KRAS activation	7	0.035	1.66E−03	5.18E−03
HALLMARK_UV_RESPONSE_UP	158	Genes upregulated in response to ultraviolet (UV) radiation	6	0.038	2.35E−03	6.90E−03
HALLMARK_CHOLESTEROL_HOMEOSTASIS	74	Genes involved in cholesterol homeostasis	4	0.0541	3.63E−03	1.01E−02
HALLMARK_WNT_BETA_CATENIN_SIGNALING	42	Genes upregulated by activation of WNT signaling through accumulation of beta catenin CTNNB1 (GeneID = 1499)	3	0.0714	5.39E−03	1.42E−02
HALLMARK_ESTROGEN_RESPONSE_EARLY	200	Genes defining early response to estrogen	6	0.03	7.35E−03	1.67E−02
HALLMARK_HYPOXIA	200	Genes upregulated in response to low oxygen levels (hypoxia)	6	0.03	7.35E−03	1.67E−02
HALLMARK_P53_PATHWAY	200	Genes involved in p53 pathways and networks	6	0.03	7.35E−03	1.67E−02
HALLMARK_REACTIVE_OXYGEN_SPECIES_PATHWAY	49	Genes upregulated by reactive oxigen species (ROS)	3	0.0612	8.29E−03	1.80E−02
HALLMARK_DNA_REPAIR	150	Genes involved in DNA repair	5	0.0333	9.18E−03	1.91E−02
HALLMARK_ANDROGEN_RESPONSE	101	Genes defining response to androgens	4	0.0396	1.08E−02	2.16E−02
HALLMARK_FATTY_ACID_METABOLISM	158	Genes encoding proteins involved in metabolism of fatty acids	5	0.0316	1.13E−02	2.18E−02
HALLMARK_BILE_ACID_METABOLISM	112	Genes involve in metabolism of bile acids and salts	4	0.0357	1.53E−02	2.83E−02
HALLMARK_APICAL_JUNCTION	200	Genes encoding components of apical junction complex	5	0.025	2.81E−02	4.84E−02
HALLMARK_ESTROGEN_RESPONSE_LATE	200	Genes defining late response to estrogen	5	0.025	2.81E−02	4.84E−02

### RNA sequencing

**FIGURE 6:**
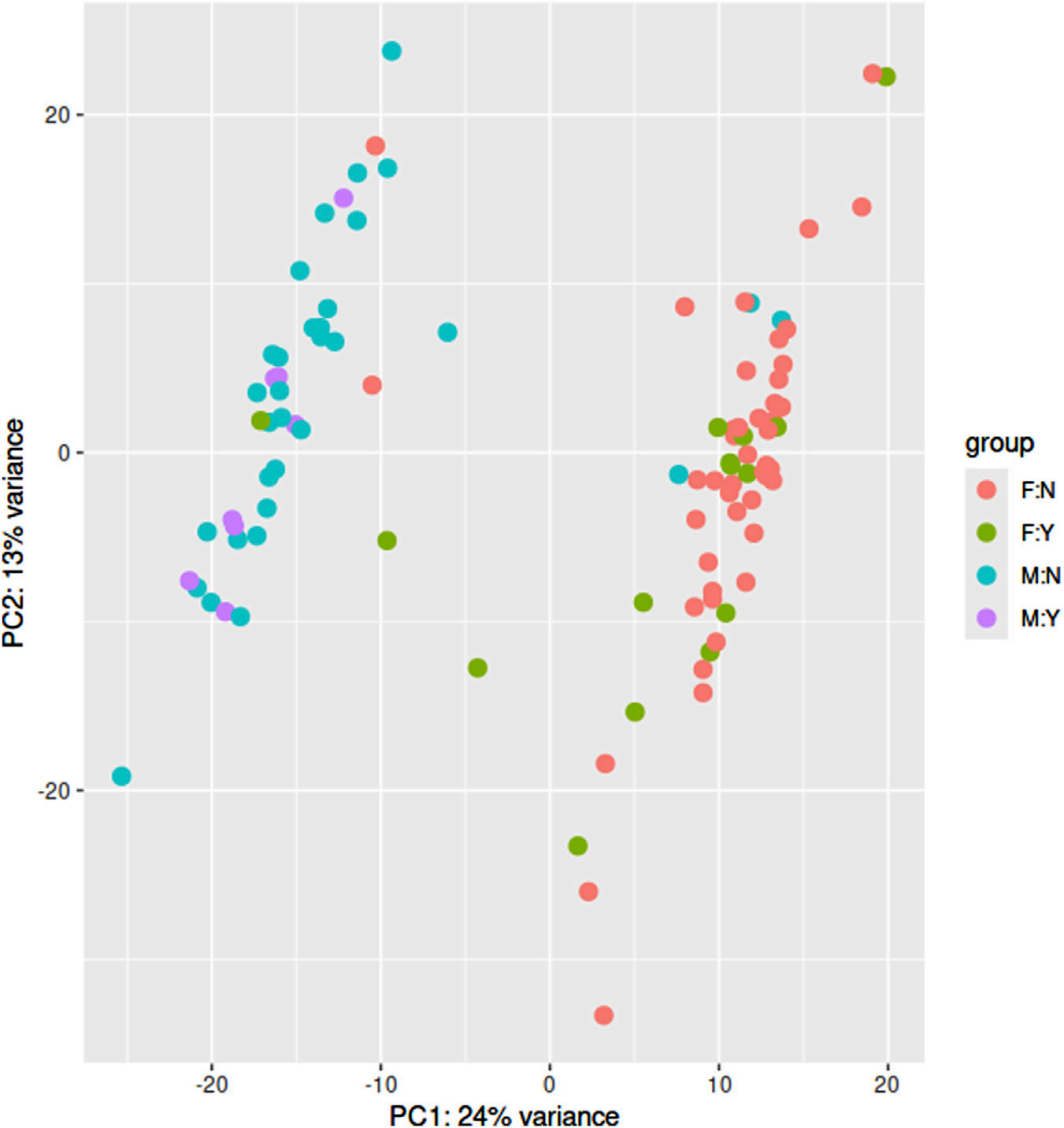
Principal component analysis of the RNA Seq data from the GEO dataset GSE247998. F:N = Female, healthy Control, F:Y = Female, Suicide Attempt, M:N = Male, healthy Control, M:Y = Male, Suicide Attempt.

To test whether we could reproduce our results on a previously published dataset, we used data from Sun et al. (2024), who compared brain and blood transcriptome profiles with respect to common genetic pathways in suicidal ideation and suicide. Since the authors of this study did not publish differential expression data on their samples, we analyzed the count data as provided in the corresponding GEO dataset using the R-package DeSeq 2. Surprisingly, we see only a small set of six genes (one pseudogene, one lncRNA and four genes related to hemoglobin biosynthesis) when comparing males with suicide attempt (SA, eight samples) to male healthy controls (11 samples) and not a single gene (|FC| > 1.5x, FDR < 0.05) when comparing females with SA (15 samples) to female healthy controls (15 samples). On the other hand, we found 439 differentially expressed genes when comparing the female suicide attempter group with the male suicide attempter group. This striking difference is also visible in a PCA plot of the samples (Figure 6), which clearly separates these two groups but not the sex-specific samples with or without SA. Since this study by Sun et al. was focused on a gene network approach we extracted the genes of the 18 significantly enriched modules from their Ingenuity Pathway analysis (IPA, data available in the supplement to this study) and used this set of 450 genes for a GSEA to identify possible overlaps with our data from the analysis of the DE genes within the HALLMARK collection. As it turned out, the overlap consisted of three genesets, namely, INFLAMMATORY_RESPONSE, COMPLEMENT and APOPTOSIS.

When we searched for corresponding GO groups within the IPA data using DAVID (Huang et al., 2009; Sherman et al., 2022), the results were similar to our own data, with the highest scoring GO groups containing “positive regulation of NF-kappaB transcription factor activity”, “positive regulation of inflammatory response”, or “positive regulation of interleukin 1 beta production”. Thus, despite the fact that we could not reproduce the sex-specific differences in gene expression between suicidal patients and healthy individuals, the differential gene expression between male and female subjects with suicide attempts leads to the same biological pathways.

## Discussion

The strong comorbidity between MDD and suicide - particularly among middle-aged men and women (Turecki and Brent, 2016) - highlights the urgent need to identify reliable risk factors. In this study, we employed Affymetrix whole-genome expression microarrays to identify key regulatory elements and pathways in the blood plasma of MDD patients with acute suicidal ideation compared to a healthy control group. Our results revealed striking sex-specific gene expression patterns, particularly involving inflammatory and NF-κB pathways, with *IL1B* (Fritz et al., 2016), *IL6* (Klawonn et al., 2021), and *TNFAIP6* among the most prominent differentially expressed genes, alongside *BEST1*, *PTPRE*, *PLAUR*, and *AQP9*.

Notably, a meta-analysis of sex-specific gene expression in blood transcriptomics of mental disorders reported TNFAIP6 as being overexpressed exclusively in females (Mohandoss and Kumar, 2023), further supporting our sex-specific findings. Several other key genes identified in our study, including *BEST1* (Ali et al., 2024), *PTPRE* (Ardesch et al., 2023), and *PLAUR* (Karagyaur et al., 2024), have been implicated in the pathophysiology of mood disorders, while *AQP9* has been proposed as a potential biomarker for both Parkinson’s disease and MDD (Wang et al., 2023). Our findings align with extensive research emphasizing the critical role of *IL6* in MDD as well as MDD-driven suicidality (Lindqvist et al., 2009), as demonstrated in both animal models (Nukina et al., 2001; Jankord et al., 2010) and clinical studies (Kern et al., 2014), and reviewed by Ting et al. (2020). Additionally, IL1B was previously identified as one of the top upstream regulators in a systems biology study by Daskalakis et al. (2024), which examined the molecular underpinnings of (PTSD) and MDD. These findings underscore the involvement of inflammatory and stress-response pathways in the molecular mechanisms underlying suicidality, particularly in females. However, while PTSD and MDD share some common gene expression patterns, they also exhibit distinct molecular signatures (Garrett et al., 2021; Jaffe et al., 2022).

Our results contribute to the growing body of evidence suggesting that immune dysregulation plays a crucial role in the pathophysiology of both major depression (Pandey et al., 2018) and suicidal behavior (Serafini et al., 2020). While several gene expression studies have been conducted [reviewed in Piras et al., (2022)], most have focused on transcriptional changes in brain tissue (Maitra et al., 2023), which is not accessible in living patients and is therefore unsuitable as a predictive biomarker source. To our knowledge, no prior study has specifically examined sex differences in gene expression patterns in acutely suicidal MDD patients.

For the first time, our exploratoy study approach revealed a substantial sex-specific discrepancy in gene expression in the blood of MDD patients with suicidal ideation. While the mixed-sex analysis identified 87 differentially expressed genes, a sex-stratified analysis revealed no differentially expressed genes among male patients, whereas 665 genes were differentially expressed in females. Despite these striking differences, our findings align closely with previous studies on the molecular biology of suicidal ideation.

Although none of the top 10 up- or downregulated genes in our dataset have been previously described in the context of suicidal ideation - possibly due to the confounding effect of combining male and female subjects - a Gene Ontology enrichment analysis revealed a significant overrepresentation of genes associated with immune system processes and inflammation. This is in accordance with prior studies in the field (Serafini et al., 2020; Piras et al., 2022).

When comaring our work to Piras et al. (2022), it is important to note, however, that only two of the studies included in the meta-analysis compared individuals with MDD driven suicide to healthy control groups, while the remaining studies included participants with other psychiatric disorders, such as schizophrenia or borderline personality disorder, which exhibit distinct DEG profiles. Still such a comparison of our differentially expressed genes with those identified in the meta-analysis by Piras et al. (2022) on suicide-related gene expression profiles highlighted two particularly interesting genes, *KCNJ2* and *A2M*. *KCNJ2* (Potassium Inwardly Rectifying Channel Subfamily J Member 2) belongs to a family of potassium channel genes implicated in various physiological responses. Emerging evidence suggests that dysregulation of potassium channel genes plays a significant role in MDD (Zhang et al., 2024a). Lai et al. (2024) demonstrated that in a human brain organoid model of traumatic brain injury, suppression of *KCNJ2* mitigates neurodegenerative processes. This raises the possibility that the observed upregulation of *KCNJ2* in our study may reflect underlying neurodegenerative mechanisms. Moreover, our analysis identified another member of the inwardly rectifying potassium channel family, *KCNJ15*, as upregulated in suicidal females with MDD. This finding may have important therapeutic implications, given that potassium channel openers have been explored as potential antidepressant treatments (Costi et al., 2022).

The second gene of interest, *A2M* (alpha-2-Macroglobulin), encodes a protease inhibitor and cytokine transporter. In our study, *A2M* was found to be downregulated in acutely suicidal female patients with MDD. Given that *A2M* inhibits inflammatory mediators (Feige et al., 2008) by blocking the IL-1β/NFKB signaling pathway (Sun et al., 2023a) - a key pathway identified in our GSEA and WGCNA analyses - this downregulation may contribute to the pervasive inflammatory signature observed in suicidal individuals with MDD as a comorbid disorder.

In 2013, Le-Niculescu et al. conducted a large-scale study on blood-based biomarkers for suicidality (Panagiotaropoulou et al., 2024), also using Affymetrix microarrays (U133 Plus 2.0). However, their study exclusively examined male patients diagnosed with bipolar disorder, limiting its generalizability to broader populations as well as its comparability to MDD driven suicidality (Panagiotaropoulou et al., 2024). Nonetheless, in our study, we identified an overlap of 33 genes with our differentially expressed gene list and 20 genes with our hub-gene list. Particularly noteworthy is the shared dysregulation of potassium channel genes and *IL1B*. Furthermore, a GSEA analysis of the Hallmark gene sets using the 246 differentially expressed genes from the Le-Niculescu study also revealed significant enrichment in the inflammatory response and TNF-α signaling via NF-κB pathways, reinforcing the biological consistency of our findings.

To further contextualize our results, we compared our data with two recent studies investigating suicidal ideation-associated pathways. The first study, by Sun et al. (2024), analyzed RNA data from both blood and brain samples of individuals with suicidal ideation and completed suicide, employing gene co-expression network analysis. The second study (Daskalakis et al., 2024), used a systems biology approach to investigate PTSD and MDD through multiple complementary methodologies.

Although the WGCNA analysis by Sun et al. identified key genes subsequently analyzed via Ingenuity Pathway Analysis (IPA), their approach differs from our GSEA methodology. However, both studies converge on the same overarching biological themes, particularly the involvement of inflammatory pathways. Using the 450 genes from their significant IPA pathways for a GSEA with the Hallmark gene sets, we observed a predominant enrichment in inflammatory response pathways. As the authors did not conduct a differential gene expression analysis, we performed this analysis ourselves using the publicly available GEO dataset.

In contrast to our blood based analysis, Daskalakis et al. (2024) primarily examined *postmortem* brain samples. Nonetheless, their identification of *IL1B* as a central upstream regulator mirrors our findings, where *IL1B* emerged as both a differentially expressed gene and a hub gene in our WGCNA network. Moreover, their study confirmed the relevance of immune regulatory and inflammatory pathways, which we also identified through our Gene Ontology enrichment, GSEA, and WGCNA analyses. This provides strong evidence that the molecular changes observed in peripheral blood reflect, at least in part, those occurring in the brain.

Transcriptional changes in blood and brain of suicidal patients were also reported by Mamdani and colleagues (2022), but they only analyzed a very small subset of 114 genes and compared the expression of these genes to a control group of people which died of various (including health related) reasons which may explain that we do not see any overlap with our differentially regulated genes.

In another very recent publication by Wang et al. (2025), blood samples from a very large group of MDD patiens with and without suicidal ideation were analyzed using RNA-Seq. Unfortunately the authors do not provide any lists of differentially regulated genes. They only report the results of a WGCNA analysis and as such, we can only compare the results of their GO analysis with our findings. As reported they found one significant module containing 1187 genes when comparing SI-patients with the corresponding healthy control group. When we compare the resulting significant GO enrichment groups with our own analysis, we observe an overlap in ten out of fifteen groups, namely: leukocyte activation involved in immune response (GO:0002366), cell activation involved in immune response (GO:0002263), phagocytosis (GO:0006909), leukocyte migration (GO:0050900), positive regulation of leukocyte activation (GO:0002696), positive regulation of cell activation (GO:0050867), leukocyte mediated immunity (GO:0002443), lymphocyte activation involved in immune response (GO:0002285), immune response-regulating cell surface receptor signaling pathway (GO:00027681), leukocyte cell-cell adhesion (GO:0007159).

In conclusion, our findings align with existing literature on the molecular underpinnings of MDD-associated suicidality and reinforce the importance of sex-specific analyses. As previously reviewed by Senei and Sibille (2014), sex differences in MDD have been documented in both human studies and animal models of the disorder. Additionally, these sex-specific differences influence treatment response, including differential efficacy of selective serotonin reuptake inhibitors (Kornstein et al., 2000). Given these findings, future research should prioritize sex-stratified analyses to refine our understanding of the molecular mechanisms underlying suicidality linked to MDD and optimize therapeutic interventions accordingly.

With respect to the underlying pathway disturbances, the present work further underlines the scientific well-founded influence of inflammatory and immune-modulating pathways on MDD, and expands this knowledge by adding novel potential biomarkers such as *TNFAIP6*.

### Limitations

It is not possible to rule out the possibility that our findings are influenced by the relatively small sample size in this study. We anticipate that differentially expressed genes will also be identified in male participants; however, these differences may be less pronounced than those observed in females. Therefore, we strongly encourage researchers conducting similar studies to assess the reproducibility of these findings by incorporating sex-specific analyses within their datasets.

Alternatively, the observed discrepancy between male and female patients may be attributable to the composition of our sample group and could stem from genetic variability among individuals, which may obscure the detection of statistically significant expression changes. This increased variability likely has a comparatively smaller impact on gene set enrichment analysis, potentially explaining the consistent findings in pathway-level analyses. Nevertheless, if this is the case, our results underscore not only the importance of considering sex-specific differences in psychiatric research but also highlight the need for a more personalized approach to patient care, moving away from the current one-size-fits-all model.

Additionally, because adverse childhood experiences (ACEs) are associated with depression and have been shown to influence gene expression (Parel and Peña, 2022), we cannot rule out their potential impact on our findings, as ACEs were not assessed in this study.

Finally, this study refrains from reporting specific biomarkers for suicidality, as it does not include a comparison group of MDD patients who are not acutely suicidal, which represents a notable limitation. Nevertheless, its primary and well-founded contribution lies in highlighting the striking sex differences observed, thereby encouraging future research to confirm whether some of the identified genes may serve as genuine sex-specific biomarkers of suicidality.

## Conclusion

This study indicates sex-specific gene expression differences between male and female patients with suicidal ideation and MDD. Hence, it may appear important to exercise caution when analyzing these patients as a single group in the future. It might not only impede the molecular findings, but also engender therapeutic differences if a gene or set of genes is only up- or downregulated in male or female patients exclusively.

### Supplemental information

Supplementary Tables S1–S3 present lists of differentially expressed genes (DEGs) identified through transcriptomic analysis. Table 1 includes DEGs (p < 0.05, FDR < 0.1, |FC| > 1.5x) in the combined male and female cohort compared to healthy controls, while Table 2 focuses specifically on DEGs within the female subset and Table 3 lists the DEGs between the male and female samples with suicide attempts. Supplementary Table S4 lists Gene Ontology (GO) categories enriched among DEGs in the female subgroup, as identified via GoMiner analysis. Supplementary Tables S5–S7 detail the leading-edge genes from gene set enrichment analysis (GSEA) for three hallmark pathways: *TNFA_SIGNALING_VIA_NFKB*, *IL6_JAK_STAT3_SIGNALING*, and *INFLAMMATORY_RESPONSE*. Supplementary Table S8 lists hub genes (Gene Significance >0.6, Module Membership >0.8) derived from Weighted Gene Co-expression Network Analysis (WGCNA) of the female sample group. Supplementary Figures S1 and S5 display high resolution STRING network analyses of DEGs in female suicide attempters (vs controls) and hub genes within the highly significant WGCNA “turquoise” module, respectively. Supplementary Figures S2–S4 provide enrichment plots for the hallmark pathways examined in GSEA: *TNFA_SIGNALING_VIA_NFKB*, *IL6_JAK_STAT3_SIGNALING*, and *INFLAMMATORY_RESPONSE*.

## Data Availability

The data presented in this study are deposited in the Gene Expression Omnibus repository (www.ncbi.nlm.nih.gov/geo), accession number GSE291874.

## References

[B1] Ali D. N. Ali H. M. Lopez M. R. Kang S. Choi D.-S. (2024). Astrocytic GABAergic regulation in alcohol use and major depressive disorders. Cells 13, 318. 10.3390/cells13040318 38391931 PMC10887002

[B2] Ardesch D. J. Libedinsky I. Scholtens L. H. Wei Y. van den Heuvel M. P. (2023). Convergence of brain transcriptomic and neuroimaging patterns in Schizophrenia, bipolar disorder, autism spectrum disorder, and major depressive disorder. Psychiatry Cogn. Neurosci. Neuroimaging 8, 630–639. 10.1016/j.bpsc.2022.12.013 37286292

[B3] Benjamini Y. Hochberg Y. (1995). Controlling the false discovery rate: a practical and powerful approach to multiple testing. J. R. Stat. Soc. Ser. B Methodol. 57, 289–300. 10.1111/j.2517-6161.1995.tb02031.x

[B4] Branney P. White A. (2008). Big boys don’t cry: depression and men. Adv. Psychiatr. Treat. 14, 256–262. 10.1192/apt.bp.106.003467

[B5] Brody D. J. Pratt L. A. Hughes J. P. (2018). Prevalence of depression among adults aged 20 and over: united States, 2013-2016. Hyattsville, MD: NCHS Data Brief, 1–8. 29638213

[B6] Castillo-Avila R. G. Genis-Mendoza A. D. Juárez-Rojop I. E. López-Narváez M. L. Dionisio-García D. M. Nolasco-Rosales G. A. (2022). High serum levels of IL-6 are associated with suicide attempt but not with high lethality suicide attempts: a preliminary case–control study. Int. J. Environ. Res. Public. Health 19, 14735. 10.3390/ijerph192214735 36429454 PMC9690459

[B7] Costi S. Han M.-H. Murrough J. W. (2022). The potential of KCNQ potassium channel openers as novel antidepressants. CNS Drugs 36, 207–216. 10.1007/s40263-021-00885-y 35258812

[B8] Daskalakis N. P. Iatrou A. Chatzinakos C. Jajoo A. Snijders C. Wylie D. (2024). Systems biology dissection of PTSD and MDD across brain regions, cell types, and blood. Science 384, eadh3707. 10.1126/science.adh3707 38781393 PMC11203158

[B9] Dudoit S. Fridlyand J. Speed T. P. (2002). Comparison of discrimination methods for the classification of tumors using gene expression data. J. Am. Stat. Assoc. 97, 77–87. 10.1198/016214502753479248

[B10] Feige J.-J. Negoescu A. Keramidas M. Souchelnitskiy S. Chambaz E. M. (2008). Alpha 2-macroglobulin: a binding protein for transforming growth factor-beta and various cytokines. Horm. Res. 45, 227–232. 10.1159/000184793 8964589

[B11] Fritz M. Klawonn A. M. Nilsson A. Singh A. K. Zajdel J. Wilhelms D. B. (2016). Prostaglandin-dependent modulation of dopaminergic neurotransmission elicits inflammation-induced aversion in mice. J. Clin. Invest. 126, 695–705. 10.1172/JCI83844 26690700 PMC4731170

[B12] Fritz M. Klawonn A. M. Jaarola M. Engblom D. (2018). Interferon-ɣ mediated signaling in the brain endothelium is critical for inflammation-induced aversion. Brain. Behav. Immun. 67, 54–58. 10.1016/j.bbi.2017.08.020 28864260

[B13] Garrett M. E. Qin X. J. Mehta D. Dennis M. F. Marx C. E. Grant G. A. (2021). Gene expression analysis in three posttraumatic stress disorder cohorts implicates inflammation and innate immunity pathways and uncovers shared genetic risk with major depressive disorder. Front. Neurosci. 15, 678548. 10.3389/fnins.2021.678548 34393704 PMC8358297

[B14] Huang D. W. Sherman B. T. Lempicki R. A. (2009). Systematic and integrative analysis of large gene lists using DAVID bioinformatics resources. Nat. Protoc. 4, 44–57. 10.1038/nprot.2008.211 19131956

[B15] Irizarry R. A. Hobbs B. Collin F. Beazer-Barclay Y. D. Antonellis K. J. Scherf U. (2003). Exploration, normalization, and summaries of high density oligonucleotide array probe level data. Biostat. Oxf. Engl. 4, 249–264. 10.1093/biostatistics/4.2.249 12925520

[B16] Iturria-Medina Y. Khan A. F. Adewale Q. Shirazi A. H. Alzheimer’s Disease Neuroimaging Initiative (2020). Blood and brain gene expression trajectories mirror neuropathology and clinical deterioration in neurodegeneration. Brain 143, 661–673. 10.1093/brain/awz400 31989163 PMC7009530

[B17] Jaffe A. E. Tao R. Page S. C. Maynard K. R. Pattie E. A. Nguyen C. V. (2022). Decoding shared Versus divergent transcriptomic signatures across cortico-amygdala circuitry in PTSD and depressive disorders. Am. J. Psychiatry 179, 673–686. 10.1176/appi.ajp.21020162 35791611 PMC10697016

[B18] Jankord R. Zhang R. Flak J. N. Solomon M. B. Albertz J. Herman J. P. (2010). Stress activation of IL-6 neurons in the hypothalamus. Am. J. Physiol. Regul. Integr. Comp. Physiol. 299, R343–R351. 10.1152/ajpregu.00131.2010 20427720 PMC2904148

[B19] Karagyaur M. Primak A. Bozov K. Sheleg D. Arbatsky M. Dzhauari S. (2024). Novel missense variants in brain morphogenic genes associated with depression and schizophrenia. Front. Psychiatry 15, 1338168. 10.3389/fpsyt.2024.1338168 38699454 PMC11063365

[B20] Kelly M. M. Tyrka A. R. Price L. H. Carpenter L. L. (2008). Sex differences in the use of coping strategies: predictors of anxiety and depressive symptoms. Depress Anxiety 25 (10), 839–846. 10.1002/da.20341 17603810 PMC4469465

[B21] Kern S. Skoog I. Börjesson-Hanson A. Blennow K. Zetterberg H. Ostling S. (2014). Higher CSF interleukin-6 and CSF interleukin-8 in current depression in older women. Results from a population-based sample. Brain. Behav. Immun. 41, 55–58. 10.1016/j.bbi.2014.05.006 24858658

[B22] Klawonn A. M. Fritz M. Castany S. Pignatelli M. Canal C. Similä F. (2021). Microglial activation elicits a negative affective state through prostaglandin-mediated modulation of striatal neurons. Immunity 54, 225–234.e6. 10.1016/j.immuni.2020.12.016 33476547

[B23] Kornstein S. G. Schatzberg A. F. Thase M. E. Yonkers K. A. McCullough J. P. Keitner G. I. (2000). Gender differences in chronic major and double depression. J. Affect. Disord. 60, 1–11. 10.1016/S0165-0327(99)00158-5 10940442

[B24] Lai J. D. Berlind J. E. Fricklas G. Lie C. Urenda J.-P. Lam K. (2024). KCNJ2 inhibition mitigates mechanical injury in a human brain organoid model of traumatic brain injury. Cell Stem Cell 31, 519–536.e8. 10.1016/j.stem.2024.03.004 38579683

[B25] Langfelder P. Horvath S. (2008). WGCNA: an R package for weighted correlation network analysis. BMC Bioinforma. 9, 559. 10.1186/1471-2105-9-559 19114008 PMC2631488

[B26] LeGates T. A. Kvarta M. D. Thompson S. M. (2019). Sex differences in antidepressant efficacy. Neuropsychopharmacology 44, 140–154. 10.1038/s41386-018-0156-z 30082889 PMC6235879

[B27] Liang W. Sun F. Zhao Y. Shan L. Lou H. (2020). Identification of susceptibility modules and genes for cardiovascular disease in diabetic patients using WGCNA analysis. J. Diabetes Res. 2020, 4178639. 10.1155/2020/4178639 32455133 PMC7238331

[B28] Lindqvist D. Janelidze S. Hagell P. Erhardt S. Samuelsson M. Minthon L. (2009). Interleukin-6 is elevated in the cerebrospinal fluid of suicide attempters and related to symptom severity. Biol. Psychiatry, Reward Dysfunct. Depress. 66, 287–292. 10.1016/j.biopsych.2009.01.030 19268915

[B29] Lopes-Ramos C. M. Chen C.-Y. Kuijjer M. L. Paulson J. N. Sonawane A. R. Fagny M. (2020). Sex differences in gene expression and regulatory networks across 29 human tissues. Cell Rep. 31, 107795. 10.1016/j.celrep.2020.107795 32579922 PMC7898458

[B30] Love M. I. Huber W. Anders S. (2014). Moderated estimation of fold change and dispersion for RNA-seq data with DESeq2. Genome Biol. 15, 550. 10.1186/s13059-014-0550-8 25516281 PMC4302049

[B31] Maitra M. Mitsuhashi H. Rahimian R. Chawla A. Yang J. Fiori L. M. (2023). Cell type specific transcriptomic differences in depression show similar patterns between males and females but implicate distinct cell types and genes. Nat. Commun. 14, 2912. 10.1038/s41467-023-38530-5 37217515 PMC10203145

[B32] Mamdani F. Weber M. D. Bunney B. Burke K. Cartagena P. Walsh D. (2022). Identification of potential blood biomarkers associated with suicide in major depressive disorder. Transl. Psychiatry 12, 159. 10.1038/s41398-022-01918-w 35422091 PMC9010430

[B33] Marcus S. M. Young E. A. Kerber K. B. Kornstein S. Farabaugh A. H. Mitchell J. (2005). Gender differences in depression: findings from the STAR*D study. J. Affect. Disord. 87, 141–150. 10.1016/j.jad.2004.09.008 15982748

[B34] Mariani N. Cattane N. Pariante C. Cattaneo A. (2021). Gene expression studies in depression development and treatment: an overview of the underlying molecular mechanisms and biological processes to identify biomarkers. Transl. Psychiatry 11, 354–23. 10.1038/s41398-021-01469-6 34103475 PMC8187383

[B35] Moderie C. Nuñez N. Fielding A. Comai S. Gobbi G. (2022). Sex differences in responses to antidepressant augmentations in treatment-resistant depression. Int. J. Neuropsychopharmacol. 25, 479–488. 10.1093/ijnp/pyac017 35167671 PMC9211005

[B36] Mohandoss A. A. Kumar S. A. K. (2023). Meta-analysis of differential expression of sex-biased and common drug metabolism enzymes and transporters genes in blood transcriptomics of mental disorders: an: in silico: study. J. Curr. Res. Sci. Med. 9, 113. 10.4103/jcrsm.jcrsm_6_23

[B37] Mootha V. K. Lindgren C. M. Eriksson K.-F. Subramanian A. Sihag S. Lehar J. (2003). PGC-1alpha-responsive genes involved in oxidative phosphorylation are coordinately downregulated in human diabetes. Nat. Genet. 34, 267–273. 10.1038/ng1180 12808457

[B38] Nukina H. Sudo N. Aiba Y. Oyama N. Koga Y. Kubo C. (2001). Restraint stress elevates the plasma interleukin-6 levels in germ-free mice. J. Neuroimmunol. 115, 46–52. 10.1016/s0165-5728(01)00260-0 11282153

[B39] Oliffe J. L. Ogrodniczuk J. S. Gordon S. J. Creighton G. Kelly M. T. Black N. (2016). Stigma in Male depression and suicide: a Canadian sex comparison study. Health J. 52, 302–310. 10.1007/s10597-015-9986-x 26733336 PMC4805721

[B40] Panagiotaropoulou G. Hellberg K.-L. G. Coleman J. R. I. Seok D. Kalman J. Mitchell P. B. (2024). Identifying genetic differences between bipolar disorder and major depression through multiple GWAS. medRxiv., 2024.01.29.24301816. 10.1101/2024.01.29.24301816 39806801 PMC12337998

[B41] Pandey G. N. Rizavi H. S. Zhang H. Bhaumik R. Ren X. (2018). Abnormal protein and mRNA expression of inflammatory cytokines in the prefrontal cortex of depressed individuals who died by suicide. J. Psychiatry Neurosci. JPN 43, 376–385. 10.1503/jpn.170192 30371993 PMC6203549

[B42] Parel S. T. Peña C. J. (2022). Genome-wide signatures of early-life stress: influence of sex. Biol. Psychiatry 91 (1), 36–42. 10.1016/j.biopsych.2020.12.010 33602500 PMC8791071

[B43] Piras I. S. Huentelman M. J. Pinna F. Paribello P. Solmi M. Murru A. (2022). A review and meta-analysis of gene expression profiles in suicide. Eur. Neuropsychopharmacol. 56, 39–49. 10.1016/j.euroneuro.2021.12.003 34923210

[B44] Radmacher M. D. McShane L. M. Simon R. (2002). A paradigm for class prediction using gene expression profiles. J. Comput. Biol. J. Comput. Mol. Cell Biol. 9, 505–511. 10.1089/106652702760138592 12162889

[B45] Ramaswamy S. Tamayo P. Rifkin R. Mukherjee S. Yeang C. H. Angelo M. (2001). Multiclass cancer diagnosis using tumor gene expression signatures. Proc. Natl. Acad. Sci. U. S. A. 98, 15149–15154. 10.1073/pnas.211566398 11742071 PMC64998

[B46] Reay W. R. Kiltschewskij D. J. Geaghan M. P. Atkins J. R. Carr V. J. Green M. J. (2022). Genetic estimates of correlation and causality between blood-based biomarkers and psychiatric disorders. Sci. Adv. 8, eabj8969. 10.1126/sciadv.abj8969 35385317 PMC8986101

[B47] Seney M. L. Sibille E. (2014). Sex differences in mood disorders: perspectives from humans and rodent models. Biol. Sex. Differ. 5, 17. 10.1186/s13293-014-0017-3 25520774 PMC4268901

[B48] Serafini G. Parisi V. M. Aguglia A. Amerio A. Sampogna G. Fiorillo A. (2020). A specific inflammatory profile underlying suicide risk? Systematic review of the main literature findings. Int. J. Environ. Res. Public. Health 17, 2393. 10.3390/ijerph17072393 32244611 PMC7177217

[B49] Sherman B. T. Hao M. Qiu J. Jiao X. Baseler M. W. Lane H. C. (2022). DAVID: a web server for functional enrichment analysis and functional annotation of gene lists (2021 update). Nucleic Acids Res. 50, W216–W221. 10.1093/nar/gkac194 35325185 PMC9252805

[B50] Shoib S. Kim Y. K. (2019). The frontiers of suicide. In: Advances in experimental medicine and biology, Kim, Y. K. (eds), vol 1192, Singapore: Springer, 503–517. 31705511 10.1007/978-981-32-9721-0_25

[B51] Simon R. Radmacher M. D. Dobbin K. McShane L. M. (2003). Pitfalls in the use of DNA microarray data for diagnostic and prognostic classification. JNCI J. Natl. Cancer Inst. 95, 14–18. 10.1093/jnci/95.1.14 12509396

[B52] Soravia S.-M. Gosemärker A.-K. Streb J. Dudeck M. Fritz M. (2024). Does gender-specific suicidal symptomatology exist? Initial work on a partially-novel, multi-questionnaire-based characterization of acute suicidal patients. Cogent Psychol. 11, 2328911. 10.1080/23311908.2024.2328911

[B53] Subramanian A. Tamayo P. Mootha V. K. Mukherjee S. Ebert B. L. Gillette M. A. (2005). Gene set enrichment analysis: a knowledge-based approach for interpreting genome-wide expression profiles. Proc. Natl. Acad. Sci. 102, 15545–15550. 10.1073/pnas.0506580102 16199517 PMC1239896

[B54] Sullivan P. F. Fan C. Perou C. M. (2006). Evaluating the comparability of gene expression in blood and brain. Am. J. Med. Genet. B Neuropsychiatr. Genet. 141B, 261–268. 10.1002/ajmg.b.30272 16526044

[B55] Sun C. Cao C. Zhao T. Guo H. Fleming B. C. Owens B. (2023a). A2M inhibits inflammatory mediators of chondrocytes by blocking IL-1β/NF-κB pathway. J. Orthop. Res. 41, 241–248. 10.1002/jor.25348 35451533 PMC12150018

[B56] Sun S. Wilson C. M. Alter S. Ge Y. Hazlett E. A. Goodman M. (2023b). Association of interleukin-6 with suicidal ideation in veterans: a longitudinal perspective. Front. Psychiatry 14, 1231031. 10.3389/fpsyt.2023.1231031 37779624 PMC10540304

[B57] Sun S. Liu Q. Wang Z. Huang Y. Sublette M. E. Dwork A. J. (2024). Brain and blood transcriptome profiles delineate common genetic pathways across suicidal ideation and suicide. Mol. Psychiatry 29, 1417–1426. 10.1038/s41380-024-02420-z 38278992 PMC11189724

[B58] Szklarczyk D. Kirsch R. Koutrouli M. Nastou K. Mehryary F. Hachilif R. (2023). The STRING database in 2023: protein-protein association networks and functional enrichment analyses for any sequenced genome of interest. Nucleic Acids Res. 51, D638–D646. 10.1093/nar/gkac1000 36370105 PMC9825434

[B59] Ting E.Y.-C. Yang A. C. Tsai S.-J. (2020). Role of Interleukin-6 in depressive disorder. Int. J. Mol. Sci. 21, 2194. 10.3390/ijms21062194 32235786 PMC7139933

[B60] Turecki G. Brent D. A. (2016). Suicide and suicidal behaviour. Lancet 387, 1227–1239. 10.1016/S0140-6736(15)00234-2 26385066 PMC5319859

[B61] Wang H. Dou S. Wang C. Gao W. Cheng B. Yan F. (2023). Identification and experimental validation of Parkinson’s disease with major depressive disorder common genes. Mol. Neurobiol. 60, 6092–6108. 10.1007/s12035-023-03451-3 37418066

[B62] Wang M. Xiang H. Wei J. Dou Y. Yan Y. Du Y. (2025). Identification of blood transcriptome modules associated with suicidal ideation in patients with major depressive disorder. Sci. Rep. 15, 1067. 10.1038/s41598-025-85431-2 39774242 PMC11706936

[B63] Weissman M. M. Bland R. C. Canino G. J. Greenwald S. Hwu H.-G. Joyce P. R. (1999). Prevalence of suicide ideation and suicide attempts in nine countries. Psychol. Med. 29, 9–17. 10.1017/S0033291798007867 10077289

[B64] Wilk A. Falk A. Joseph R. Smith S. (2023). Mood and anxiety disorders: suicide. FP Essent. 527, 19–24. 37036768

[B65] Wright G. W. Simon R. M. (2003). A random variance model for detection of differential gene expression in small microarray experiments. Bioinformatics 19, 2448–2455. 10.1093/bioinformatics/btg345 14668230

[B66] Zeeberg B. R. Feng W. Wang G. Wang M. D. Fojo A. T. Sunshine M. (2003). GoMiner: a resource for biological interpretation of genomic and proteomic data. Genome Biol. 4, R28. 10.1186/gb-2003-4-4-r28 12702209 PMC154579

[B67] Zhang B. Horvath S. (2005). A general framework for weighted gene co-expression network analysis. Stat. Appl. Genet. Mol. Biol. 4, Article17. 10.2202/1544-6115.1128 16646834

[B68] Zhang J. Zhu Y. Zhang M. Yan J. Zheng Y. Yao L. (2024a). Potassium channels in depression: emerging roles and potential targets. Cell Biosci. 14, 136. 10.1186/s13578-024-01319-0 39529121 PMC11555980

[B69] Zhang Y. Jia X. Yang Y. Sun N. Shi S. Wang W. (2024b). Change in the global burden of depression from 1990-2019 and its prediction for 2030. J. Psychiatr. Res. 178, 16–22. 10.1016/j.jpsychires.2024.07.054 39106579

[B70] Zoller V. Funcke J.-B. Roos J. Dahlhaus M. Abd El Hay M. Holzmann K. (2017). Trail (TNF-related apoptosis-inducing ligand) induces an inflammatory response in human adipocytes. Sci. Rep. 7, 5691. 10.1038/s41598-017-05932-7 28720906 PMC5515939

