# Supplementary figures and images for: Sex-specific gene expression and weighted co-expression network analysis suggest distinct sex-specific molecular signatures in acutely suicidal MDD-patients without somatic comorbidities
Source: Front Genet. 2025 Oct 3;16:1653768. doi: 10.3389/fgene.2025.1653768 (PMC12531222; doi:10.3389/fgene.2025.1653768)

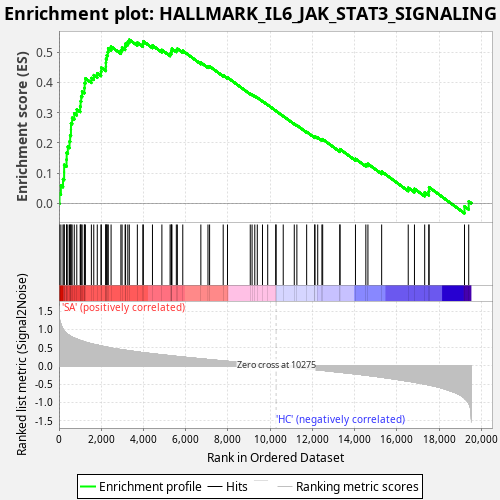

Supplement: Supplementary file 2 [file Image3.jpeg]

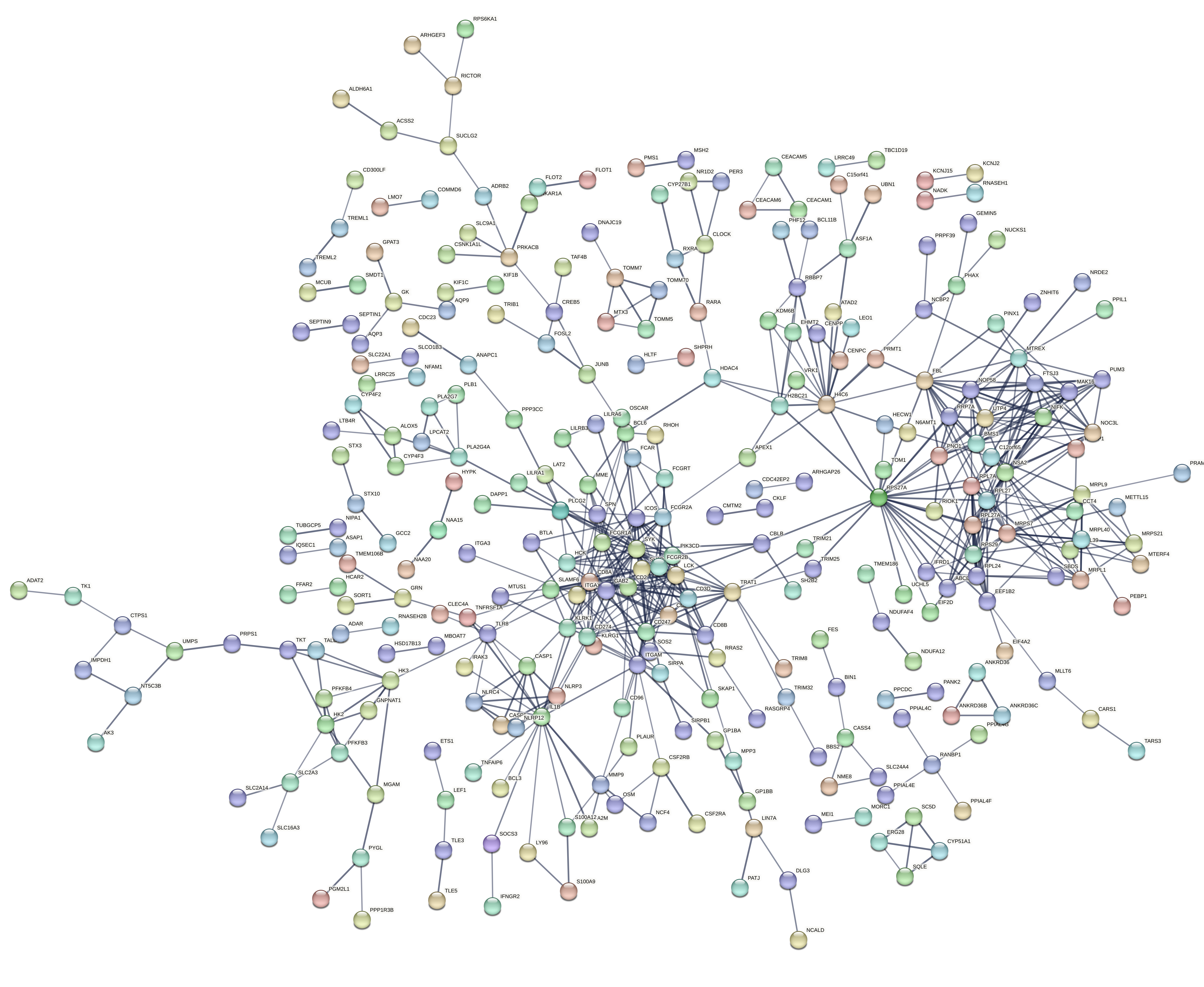

Supplement: Supplementary file 4 [file Image1.jpeg]

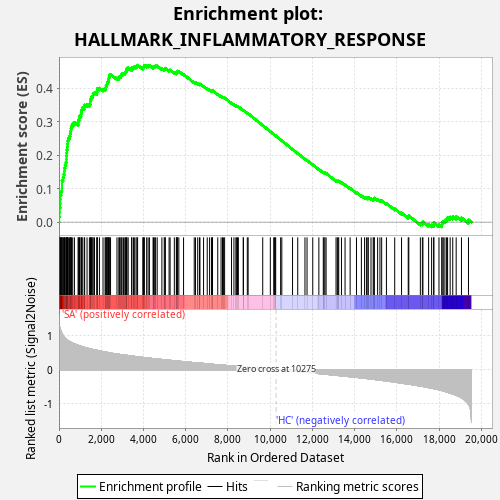

Supplement: Supplementary file 5 [file Image4.jpeg]

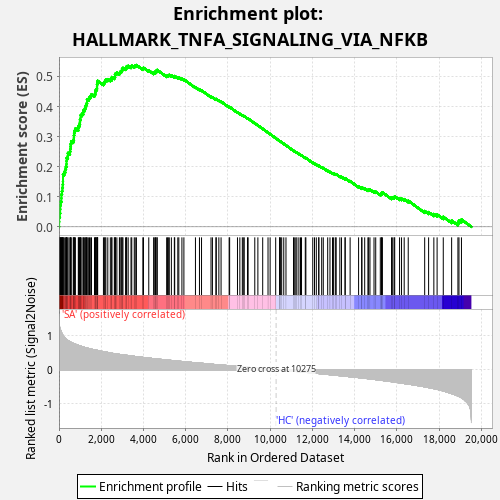

Supplement: Supplementary file 6 [file Image2.jpeg]

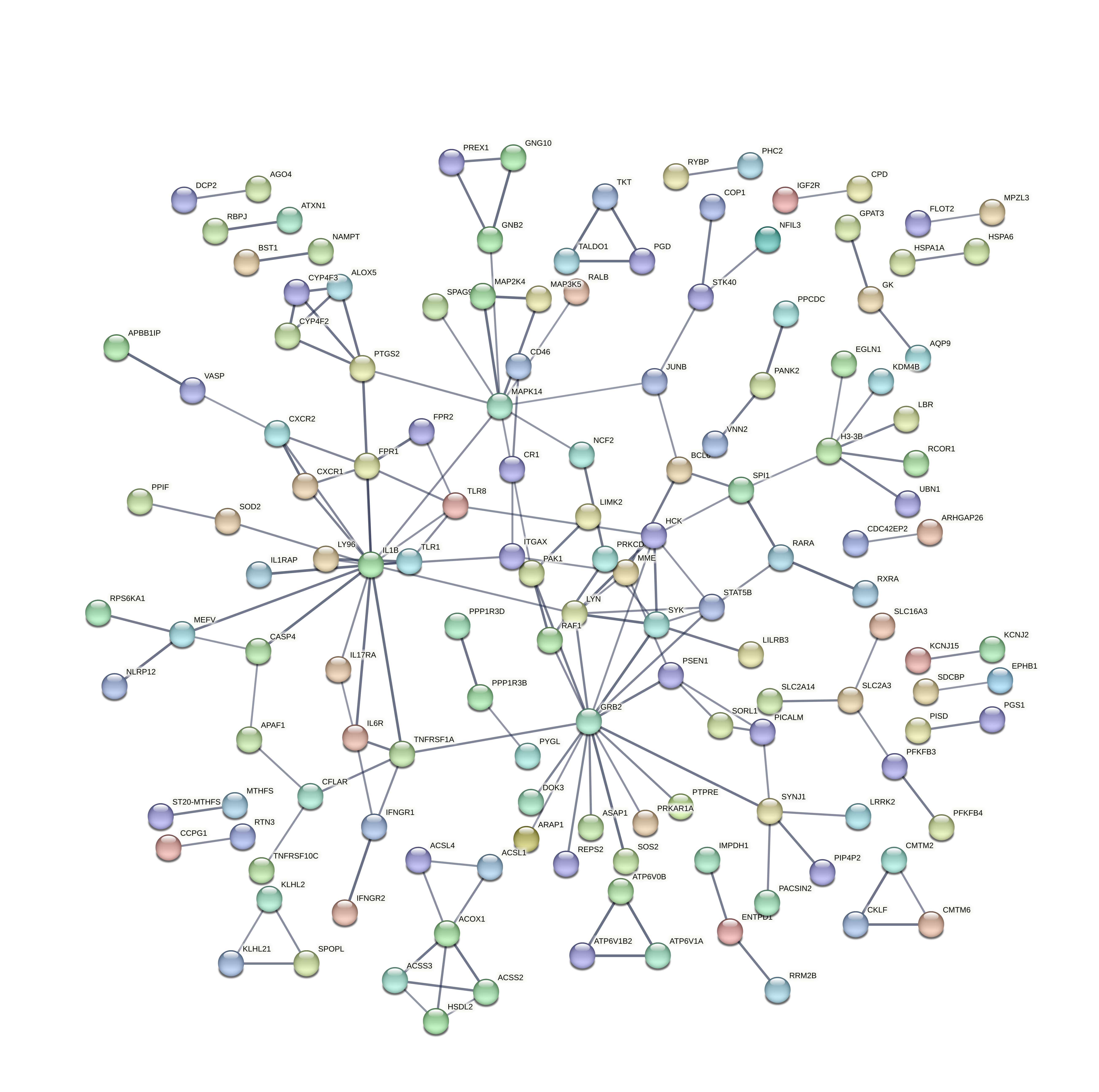

Supplement: Supplementary file 7 [file Image5.jpeg]
